# Predictors of response to electroconvulsive therapy and its importance in the treatment of patients with obsessive-compulsive disorder

**DOI:** 10.1192/j.eurpsy.2021.1310

**Published:** 2021-08-13

**Authors:** J. Martins Correia, M.I. Fonseca Marinho Vaz Soares, S. Freitas Ramos, B. Jesus, D. Cruz E Sousa, S. Caetano

**Affiliations:** Department Of Psychiatry And Mental Health, Local Health Unit of Guarda, Guarda, Portugal

**Keywords:** obessive-compulsive disorder, Electroconvulsive therapy

## Abstract

**Introduction:**

Electroconvulsive therapy (ECT) presents itself as a highly effective therapeutic approach in various psychiatric conditions, especially affective disorders and catatonia. Although obsessive-compulsive disorder (OCD) is not an established indication for ECT, there are several positive results that have been replicated, giving us an account of its potential applicability.

**Objectives:**

To emphasize the importance of defining predictors of response to ECT in OCD.

**Methods:**

The authors’ clinical experience is combined with the review of clinical cases, available in the literature, related to the application of ECT in OCD.

**Results:**

Personal or family history of affective pathology and obsessions of sexual content were identified as potential predictors of response to ECT in patients with obsessive and compulsive symptoms.

**Conclusions:**

Although preliminary and based solely on case reports, the replicability of results should promote special attention to situations in which OCD is marked by particular characteristics that favor the response to ECT. In this way, it would be possible to prevent the dragged consumption of health resources and minimize the expected chronicity associated with this clinical condition.

